# Understanding the mechanisms that facilitate specificity, not redundancy, of chemokine‐mediated leukocyte recruitment

**DOI:** 10.1111/imm.13200

**Published:** 2020-05-06

**Authors:** Douglas P. Dyer

**Affiliations:** ^1^ Wellcome Centre for Cell‐Matrix Research Lydia Becker Institute of Immunology and Inflammation Faculty of Biology, Medicine and Health Manchester Academic Health Science Centre University of Manchester Manchester UK

**Keywords:** chemokine/chemokine receptors, chemokines, chemotaxis, inflammation

## Abstract

Chemokines (chemotactic cytokines) and their receptors are critical to recruitment and positioning of cells during development and the immune response. The chemokine system has long been described as redundant for a number of reasons, where multiple chemokine ligands can bind to multiple receptors and vice versa. This apparent redundancy has been thought to be a major reason for the failure of drugs targeting chemokines during inflammatory disease. We are now beginning to understand that chemokine biology is in fact based around a high degree of specificity, where each chemokine and receptor plays a particular role in the immune response. This specificity hypothesis is supported by a number of recent studies designed to address this problem. This review will detail these studies and the mechanisms that produce this specificity of function with an emphasis on the emerging role of chemokine–glycosaminoglycan interactions.

## Graphical abstract

Chemokines (chemotactic cytokines) and their receptors are critical to recruitment and positioning of cells during development and the immune response. Supposed redundancy of the chemokine system has been thought to be a major reason for the failure of drugs targeting chemokines during inflammatory disease. We are now beginning to understand that chemokine biology is in fact based around a high degree of specificity, where each chemokine and receptor plays a particular role in the immune response.

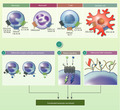

**Abbreviations**
C‐2: carbon 2 position
GAG: glycosaminoglycan
GlcA: glucuronic acid
GlcNAc: *N*‐acetyl‐d‐glucosamine
HS: heparan sulphate
iCCR: inflammatory chemokine receptors
Th2: T helper type 2

## Introduction

Chemokine ligands and their receptors facilitate the movement of leukocytes from the circulation, through the endothelium and into underlying tissues.^1^, ^2^, ^3^, ^4^, ^5^, ^6^, ^7^, ^8^ This process is critical during inflammation to enable immune‐cell‐mediated clearance of invasive pathogens, e.g. through recruitment of neutrophils, and is also critical in immune surveillance of tissues, e.g. entry of memory T cells. Chemokines and their receptors have also been shown to be critical in the movement and positioning of cells within tissues.

Chemokines are defined by their structure and show a high level of quaternary structural similarity.^1^, ^2^, ^3^, ^4^, ^5^, ^6^, ^7^, ^8^ They can be split into families based on the separation of their first two cysteine residues comprising the CC family (no separation), CXC (separated by one amino acid), CX3C (separated by three amino acids) and XC (with only the second cysteine residue). The latter two families are comprised of only one chemokine with the first two families being dominant containing 28 and 17 chemokine ligands, respectively.

Chemokines mediate their myriad of functions, principally cellular recruitment, by binding to their cognate receptors on cells, primarily leukocytes.^1^, ^2^, ^3^, ^4^, ^5^, ^6^, ^7^ These interactions produce signalling events leading to integrin activation, e.g. MAC‐1, VLA‐4 and LFA‐1 enabling firm adhesion to extracellular matrix ligands, e.g. ICAM‐1 and ICAM‐2, VCAM‐1 and INAM‐1.^9^, ^10^, ^11^ In addition, chemokines have biological functions beyond cellular recruitment, for example effects on cell activation, non‐leukocyte biology and secretion of signalling molecules, e.g. cytokines.^12^, ^13^, ^14^, ^15^, ^16^, ^17^, ^18^

The same mechanisms that are critical to the correct functionality of the chemokine system also facilitate inflammation during diseases such as rheumatoid arthritis and atherosclerosis as well as mechanisms underlying cancer pathogenesis, e.g. metastasis.^6^, ^19^, ^20^, ^21^, ^22^, ^23^, ^24^

Because of their central role in a healthy immune system, as well as inflammatory‐based diseases and cancer, chemokines have been the focus of translational research since their discovery 30 years ago.^25^, ^26^ There are two chemokine‐targeted therapies in the clinic: plerixafor, a CXCR4 antagonist that facilitates stem cell mobilization, and maraviroc, a CCR5 antagonist that inhibits human immunodeficiency virus entry into cells.^27^ However, we have yet to successfully target the chemokine system during inflammatory disease. A number of trials have failed for a range of different reasons such as pharmacokinetics.^25^, ^26^, ^28^ One example is the failure to produce a sufficiently high plasma concentration of drug to achieve the 90% chemokine receptor occupancy that will be required for efficacy (reviewed in ref. 25). However, the overarching issue is our lack of a holistic understanding of how the chemokine system functions during the inflammatory response.

**Figure 1:**
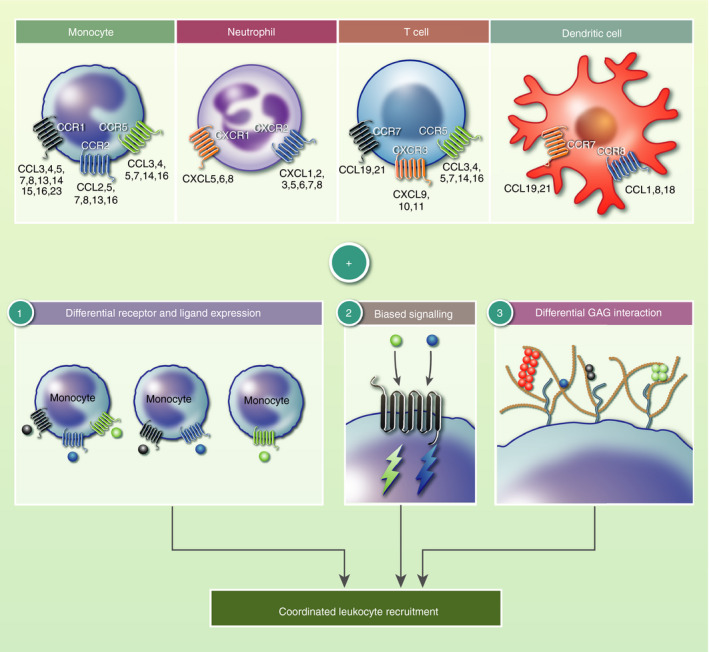
Specificity of the chemokine system and the mechanisms that produce it. Chemokine ligands can bind to various chemokine receptors and vice versa. In addition, the same receptor can be expressed by different types of immune cell. This has classically led to the idea of redundancy in the system. Detailed analysis has demonstrated that the chemokine system is in fact based around specificity of receptor and ligand function. This is produced by a number of mechanisms. (1) Differential receptor and ligand expression to localize signals. (2) Biased signalling, where different ligands can produce different signalling outcomes via the same receptor. (3) Differential interaction with glycosaminoglycans that are present within vascular and tissue extracellular matrix. Together these mechanisms facilitate specific function of each chemokine ligand and receptor during immune cell recruitment.

The central issue that has been thought to preclude targeting the chemokine system is redundancy.^25^, ^26^, ^27^ This has centred around the fact that numerous chemokines can bind to numerous receptors and vice versa (Fig. 1). However, we have begun to think that the chemokine system may actually be based on a very high degree of specificity requiring subtlety of analysis.^5^, ^25^, ^26^, ^29^, ^30^ This idea of specificity is that each chemokine ligand and receptor is playing a particular non‐redundant role in the immune response, primarily recruitment of leukocytes. A previous review by Schall and Proudfoot^25^ proposed that pharmacology issues and target selection are the dominant reasons for trial failure and not redundancy of the chemokine system. The authors proposed that the available pharmacological data actually provide further evidence of specificity rather than redundancy of the chemokine system. A number of recent papers have strengthened this idea, demonstrating specificity of function for both chemokine ligands and their receptors during the inflammatory response.

This review will describe examples of specificity and the potential mechanisms that produce it (Fig. 1), and will suggest how this knowledge may help to inform therapeutic development.

## Differential chemokine receptor and ligand expression and tissue specificity

The first and most obvious way to create specificity is differential expression of chemokines and receptors, localizing them to different sites, and stages, of leukocyte recruitment (Fig. 1). Geographical expression would also help to explain the concept of tissue‐specific roles of chemokine ligands and receptors. In addition, and/or in combination, differential expression of chemokines and receptors in certain biological scenarios, e.g. local and systemic inflammation, would also create specificity of function. Below are a number of examples of specificity of receptor and ligand function that may be explained by their differential geographical and environmental expression.

We recently demonstrated specificity of function for chemokine receptors long‐described as redundant.^31^ The inflammatory chemokine receptors Ccr1, ‐2, ‐3 and ‐5 (iCCR receptors) are clustered together in humans (chromosome 3) and mice (chromosome 9) and have been associated with expression on monocytes/macrophages (CCR1, ‐2 and ‐5), T cells (CCR1, ‐2 and ‐5) and eosinophils (CCR3).^32^, ^33^

In this study, we observed numerous examples of specificity, with CCR2 being confirmed as the dominant receptor facilitating emigration of monocytes from the bone marrow and subsequent transiting from the circulation to tissues (spleen and lung). This was confirmed in both resting and inflammatory contexts. We demonstrated that CCR1 and CCR5 may have additional specialized roles in emigration of monocytes from the bone marrow (CCR1) or entry into the spleen (CCR1 and CCR5), and speculated that they may be involved in monocyte localization within tissues.

The primary role of the iCCR receptors, defined above, is in monocyte and eosinophil recruitment during inflammation.^32^, ^33^ In three separate inflammatory models, carrageenan‐mediated air pouch recruitment, zymosan‐mediated peritonitis and influenza virus infection of the lung, specificity of receptor function was clear. Specifically, CCR2 is largely responsible for monocyte release from the bone marrow and for the majority of monocyte migration from the circulation to inflamed tissue. Surprisingly and importantly 40% of monocytes, compared with wild‐type controls, could still be recruited to the inflamed peritoneum in the absence of CCR2. Inflammatory recruitment of eosinophils was shown to be fully reliant on CCR3, as expected. Work is now ongoing to determine the function of these iCCR receptors to determine the specific roles for CCR1 and CCR5 in monocyte/macrophage biology.

This specificity of iCCR receptor function is probably explained by co‐ordinated spatial and cellular expression of these receptors, a possibility that is currently being investigated (Medina‐Ruiz and Graham, personal communication). A key aspect of this research will be to establish the expression pattern of these receptors across different tissues to determine their function in different sites around the body.

In this study, we also observed an intriguing example of tissue‐specific function of chemokine ligands. We found that monocytes use different chemokine ligands to enter different tissues, specifically CCL5 to enter the lung and CCL7 in the skin. The mechanism underlying such specificity is unclear, but the idea of tissue‐specific co‐ordination remains an appealing one for tailored therapeutics.

Hence, it seems likely that recruitment of eosinophils and monocytes is collaboratively mediated by CCR1, ‐2, ‐3, ‐5, and their ligands, with each expressed in a specific location and time in response to specific stimuli to facilitate different steps of recruitment of these cells.

Further examples of specificity within the chemokine system associated with controlled expression are the chemokine ligands CXCL9, CXCL10 and CXCL11. These ligands mediate chemotaxis of cells via their shared receptor CXCR3, usually expressed on T cells.^29^ CXCR3 has been shown to play a non‐redundant (specific) role in T‐cell trafficking to human and mouse tumours.^34^ Specificity of function has been demonstrated for CXCL9 and CXCL10 in mice; CXCL11 is not expressed at the protein level in C57/BL6 mice.^29^

CXCR3 ligand functional specificity has been examined in a biological context by Groom *et al.* ^35^ using reporter mice (REX3) with red fluorescent protein and blue fluorescent protein under the control of the CXCL9 and CXCL10 promoters, respectively. In this study, the authors demonstrate that CXCL9 and CXCL10 have non‐redundant roles in T‐cell polarization after immunization. In this context, CXCL10 expression is localized to bone‐marrow‐derived haematopoietic cells in contrast to CXCL9, which is expressed in the stromal cell compartment within the medulla and intrafollicular area of the draining lymph node. As a result, the two ligands were found to play non‐redundant roles in the polarization of T helper type 1 cells following immunization as the result of localization of T cells within the draining lymph nodes.

Similarly, CXCL9, ‐10 and ‐11 can be expressed at different times by different stimuli in other contexts. CXCL11 is the dominant ligand in transplantation‐associated inflammation,^36^ CXCL9 and CXCL10 are produced and act in spatially distinct scenarios.^35^

There is an extensive literature associated with the specific roles for CXCL9, ‐10 and ‐11 function via CXCR3 and their non‐redundant function that is revealed by intensive mechanistic analysis of their biology (extensively reviewed in ref. 37). Hence, co‐ordinated expression of CXCL9, ‐10 and ‐11 facilitates their differential role in specific steps of T‐cell recruitment and polarization.

Girbl *et al.* recently addressed the problem of chemokine redundancy in the context of murine neutrophil recruitment. This study provided further evidence for the specificity of function and not redundancy due to differential expression patterns of two murine neutrophil chemoattractants, CXCL1 and CXCL2.^38^ The authors used multi‐photon imaging of the neutrophil recruitment cascade in the mouse cremaster muscle to dissect the specific roles of these chemokines.

The authors demonstrated that neutrophil recruitment is dependent on both CXCL1 and CXCL2 via their murine receptor, CXCR2.^39^ They went on to show that these two ligands function stepwise to facilitate distinct stages of the recruitment process.^38^ Specifically, CXCL1 enabled luminal crawling of neutrophils and CXCL2 facilitated correct breaching of endothelial cell junctions, so acting in a sequential manner.

The mechanism underlying this specificity was a product of differential expression following an inflammatory stimulus with tumour necrosis factor‐*α*. CXCL1 is produced by both endothelial cells and pericytes, whereas CXCL2 was not present in these locations under the same conditions but was found to be produced by neutrophils in response to tumour necrosis factor‐*α*. In addition to specificity of expression, CXCL2 was shown to be localized to the junction of endothelial cells via presentation on the atypical chemokine receptor ACKR1. This study describes specificity, in this instance of ligand function, again due to differential expression and localization.

There are a number of additional historical examples where chemokine receptor and ligand expression may explain specificity of function. One representative example is the receptor CCR4, largely associated with T lymphocytes during the T helper type 2 (Th2) response.^40^ CCR4 has two ligands CCL17 and CCL22; specificity of function of this receptor and its ligands has been localized to the skin through expression at this site.^41^ Synthesis of analysis from a number of papers suggests that CCL17 and CCL22 are differentially expressed and localized within inflamed skin, possibly facilitating stepwise roles in recruitment of T cells in this context.^41^, ^42^, ^43^

To dissect specific chemokine function, future studies will be needed to analyse localized expression of chemokines and their receptors within biological and disease scenarios. This will enable correct therapeutic target selection for development of novel drugs.

These studies clearly demonstrate that differential expression of chemokine receptors and ligands exists in biological scenarios. However, there are many instances in biology where an array of chemokines are present that recruit the same cell and that act via the same receptors. This creates a fundamental problem of how immune cells can interpret and properly respond to such complex signals. There must, therefore, be additional mechanisms, beyond expression, that produce specificity within the chemokine system.

## Biased signalling and differential receptor function

One way in which complex chemokine signals may be interpreted by cells is through biased signalling and differential receptor function. Biased signalling is a recent development in understanding seven transmembrane receptor biology and function that underlies chemokine signalling. Biased signalling is where two different ligands can bind to the same receptor and produce different signalling and biological outcomes.^44^ Such an effect facilitates chemokine specificity as different ligands have different effects, via the same receptor, on migrating leukocytes. There is now a large body of literature describing this phenomenon in the chemokine system.

The CXCR3, CXCL9, CXCL10 and CXCL11 system exemplifies biased signalling facilitating specificity (reviewed in ref. 37). These ligands bind to different parts of CXCR3, have different affinities for the receptor and have different signalling outcomes through CXCR3, resulting in different potencies in *in vitro* chemotaxis experiments.

CCR4 is expressed in Th2 lymphocytes and induces cell migration in response to both CCL17 and CCL22 *in vitro.* ^40^, ^45^ CCL22 is the dominant ligand and prevents CCR4 from responding to CCL17; however, in the reverse experiment CCL17 did not de‐sensitize CCR4 to CCL22.^46^ CCL22 also triggers greater internalization and reduced CCR4 recycling to the surface in Th2 cells.^45^ Similarly, CCR7 has two established ligands, CCL19 and CCL21, that together co‐ordinate dendritic cell migration and T‐cell positioning.^47^, ^48^ These two ligands have been shown to have differential outcomes via their shared CCR7 receptor (reviewed in ref. 49).

A recent study described how CXCL11 and CXCL12 interact with strikingly different kinetics with their shared receptor ACKR3.^50^ This study suggests that ligand binding kinetics for their receptors may affect *β*‐arrestin recruitment to the receptor, so mediating signalling outcome and creating specificity.

Hence, there are a number of instances where different chemokine ligands produce different signalling outcomes from the same receptor. This mechanism is integral in creating specificity of cellular responses through a single receptor in the presence of different ligands (Fig. 1).

As well as biased signalling, related chemokine receptors may be expressed on the same cells but have different functional roles due to receptor behaviour. Coombs *et al.* ^51^ have recently demonstrated that CXCR1 and CXCR2 play specific roles in collaboratively co‐ordinating neutrophil migration within damaged tissue in zebrafish. They systematically dissect this process to reveal that CXCR1 controls neutrophil clustering whereas CXCR2 facilitates multi‐directional movement of neutrophils. Furthermore, CXCR2 is maintained at the plasma membrane for longer periods of time and in this way enables neutrophil movement away from sites of clustering.

A further example of signalling specificity is demonstrated by a human neutrophil chemoattractant, CXCL8, having different effects on internalization of CXCR1 and CXCR2 human neutrophil receptors, producing much more rapid internalization of CXCR2 compared with CXCR1.^52^ These different effects of the same ligand on different receptors could elicit specific outcomes according to what is temporally and geographically required for leukocyte migration.

A thorough and complete understanding of the biased signalling and receptor–ligand‐specific outcomes within the chemokine system will be vital in developing therapeutics. Chemokine receptor antagonists that have specific and predictable outcomes on cellular recruitment are likely to be powerful drugs in the clinic.

## Glycosaminoglycan interactions

**Figure 2:**
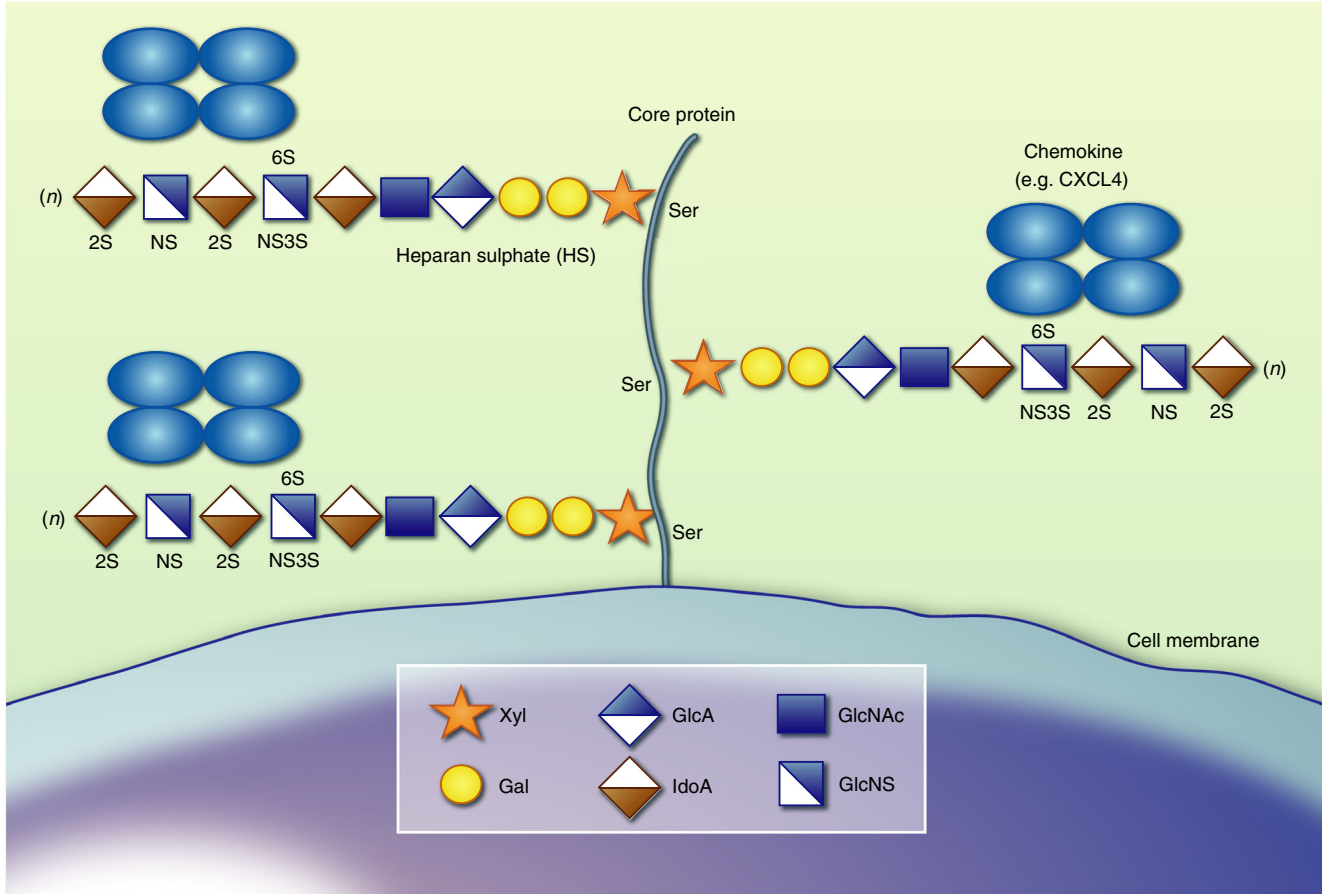
Heparan sulphate proteoglycan structure. Heparan sulphate (HS) proteoglycans have a protein core that is cell membrane embedded, as depicted here (syndecan 1–4 and glypican 1–6), or soluble (serglycin and agrin) decorated with sugar side chains. These HS sugar side chains are attached to a serine residue and have an initial linker followed by repeating disaccharide units of glucuronic acid (GlcA) and *N*‐acetyl‐d‐glucosamine (GlcNAc). GlcA can be epimerized to iduronic acid (IdoA) and sulphated at the C‐2 position. GlcNAc can be *N*‐sulphated to GlcNS, with sulphate groups also added at C‐6 and sometimes C‐3. Proteoglycans can cluster together to form a glycocalyx on different cell types. In particular, the endothelial glycocalyx, largely composed of proteoglycans, forms a barrier that controls blood vessel permeability and immune cell migration.

A further way in which complex chemokine signals may be interpreted is via differential binding and localization on the extracellular matrix within tissues and the vasculature (Fig. 1). A relatively unexplored mechanism behind specific localization and positioning of chemokine ligands is their interaction with glycosaminoglycans (GAGs) (Fig. 2).

It has been known for a number of years that the ability of chemokines to interact with GAGs, via oligomerization, is critical for the *in vivo* functionality of certain chemokines, e.g. CCL2, CCL5, CXCL8 and CXCL10.^53^, ^54^, ^55^, ^56^, ^57^, ^58^ Interfering with this interaction has been shown to inhibit chemokine‐mediated leukocyte recruitment *in vitro* and *in vivo*.^59^, ^60^, ^61^, ^62^, ^63^, ^64^, ^65^ These findings may explain some of the anti‐inflammatory effects of heparin and heparin‐related therapeutics that would disrupt chemokine–GAG interactions and therefore inhibit chemokine‐mediated leukocyte recruitment.^66^ These interactions localize chemokines and protect them from proteolysis;^67^, ^68^ however, we lack a clear understanding of the biological role of chemokine–GAG interactions.^69^ The importance of these interactions has led to efforts to target them to ameliorate chemokine‐driven inflammatory disease (reviewed in ref. 70).

GAGs are sugar chains that decorate protein cores, together termed proteoglycans (Fig. 2).^71^ Proteoglycans are found within the extracellular matrix in the basement membrane, on the majority of cell surfaces and also form the thick glycocalyx barrier that lines blood vessels and regulates leukocyte recruitment.^72^ Heparan sulphate (HS) is the dominant GAG in the context of endothelial chemokine presentation and leukocyte migration. Chondroitin sulphate and dermatan sulphate, which have slightly different sequences, may also contribute to a lesser degree. HS GAGs are made up of repeating disaccharides of glucuronic acid (GlcA) and *N*‐acetyl‐d‐glucosamine (GlcNAc) (Fig. 2).^71^ GlcA residues can be epimerized to iduronic acid and sulphated at the carbon‐2 (C‐2) position. GlcNAc residues can be *N*‐deacetylated and *N*‐sulphated, with sulphate groups also added at the C‐6 and much more rarely at the C‐3 position by a network of sulphotransferases.^73^ The HS GAGs can be further modified by endosulphatases, SULF1 and SULF2, that remove sulphate groups, heparanases that cleave the GAG chains and extracellular proteases. These modifications result in complex polysaccharides containing islands of high and low sulphation separated by non‐sulphated regions.^71^ A range of proteins, such as chemokines, bind to these islands of sulphation, it seems likely that the non‐sulphated regions contribute to these interactions, but it remains unclear how.

### Differential chemokine–GAG interactions

A new interpretation of the importance of differential chemokine–GAG interactions, demonstrated by a number of groups,^63^, ^74^, ^75^, ^76^, ^77^, ^78^, ^79^ is that they facilitate differential chemokine localization. Importantly localization would be achieved even when chemokines are produced within the same local niche, as is the case during inflammation.

The most established example of the biological importance of differential GAG binding is in relation to the CCR7 ligands, CCL19 and CCL21. Three papers from the Sixt group have shown that these two ligands achieve functional specificity due to their differential ability to bind and be presented on the extracellular matrix components, HS and polysialic acid.^47^, ^48^, ^80^ Their first paper proposed that CCL21 is immobilized to HS on the endothelial surface, where it facilitates random adhesive migration of dendritic cells.^47^ In contrast, CCL19 has a much lower affinity interaction with HS and so principally functions in a soluble form to enable directed migration of these same cells. Interestingly a cleaved soluble form of CCL21 elicited the same effects as CCL19, suggesting that immobilization itself is a key aspect of chemokine function. In a further study, this group went on to produce one of the few papers demonstrating a chemokine gradient formed by CCL21 to guide dendritic cells.^80^ Again, this gradient‐forming effect of interaction with HS differentiated CCL21 function from CCL19.

More recently the Sixt group has demonstrated a further difference in CCL21 and CCL19 function mediated through adhesion to extracellular matrix.^48^ The basic C‐terminus of CCL21, which is absent in CCL19, binds to polysialic acid, which is a post‐translational modification of the CCR7 receptor. This charge‐based interaction promotes a structural change within CCL21 that enables the chemokine to bind to CCR7. Polysialic acid is composed of acidic sugar residues and is usually found at the end of sugar chains on cells and proteins in various locations including the lymph node.^48^ Their paper demonstrated a clear role for sialic acid in regulating dendritic cell trafficking in this location alongside its varying role in immune responses.

These studies have elegantly illustrated the biological importance of differential chemokine–GAG interactions. We can now expand on this well‐studied example to explore the importance of this mechanism in producing specificity of chemokine function more widely.

In general, chemokines display a wide range of affinities for GAGs. A hierarchy of interaction has been established with the highest affinity chemokines showing oligomeric propensity and a basic charge.^75^ Using a range of chemokines in the same study we established a clear hierarchy of affinity where CXCL4, CCL5 and CXCL11 have the highest affinity followed by CXCL12 and CCL2 with intermediate affinity. CXCL8 had the weakest observable interaction of the group tested. Indeed, if we extend this to look at chemokines likely to be similarly expressed and recruit the same cell type, e.g. CXCL4, CCL2, CCL3, CCL5 and CCL7 (implicated in monocyte recruitment), we see a wide range of affinities for GAGs. Our more recent analysis suggests that this trend is widespread in systems where multiple ligands bind to the same receptor (unpublished data).

These findings suggest that in the numerous examples where complex mixtures of chemokines with overlapping receptor and cell‐binding propensities are produced, specificity can still be achieved. Differential interaction with GAGs will mean that some chemokines are retained locally whereas others will diffuse much further from their site of production, facilitating gradient formation within tissues.

### GAG fine structure facilitates specificity of chemokine binding

Recent breakthroughs in the field of GAG biology are now giving a glimpse into the high degree of specificity that GAG interactions may entail.^81^, ^82^ Chemokine–GAG interactions have been under‐estimated as being driven by ‘non‐specific’ binding. Recent publications have demonstrated fine‐tuning of the islands of sulphation on GAGs that determine these interactions (Fig. 2). Relatively subtle changes in GAG fine structure have been shown to help define which chemokines they bind to and present. Miller *et al.* ^81^ demonstrated that CCL2 has a 200‐fold difference in affinity for two hexasaccharides that differ in position of a single sulphate at either the C‐2 (2‐O sulphation) of uronic acid or C‐6 (6‐O sulphation) of glucosamine residues. Similarly, Jayson *et al.* ^82^ have shown that modification of a terminal glucosamine with a 6‐O sulphate group converts an oligosaccharide from an inhibitor of CXCL8 to an inhibitor of CXCL12 biological function. Our own work assessed the effect of removal of 2‐O sulphation from heparin on its ability to bind different chemokines. This study showed that 2‐O sulphation was differentially important for interaction with CXCL11, CXCL12, CCL2 and CCL5 while being unnecessary for binding to CXCL4.^75^ These studies provide a glimpse of the significant effects on chemokine function mediated by subtle changes in GAG fine structure. This fine‐tuning adds further credence to the ability of GAGs to play a key role in specific localization of chemokine ligands.

Traditionally heparin, a GAG only found in mast cells in biology, is often used as a surrogate for the more relevant GAG HS.^71^ Although this remains a valid approach, recent moves towards using HS, and modified versions of it, will facilitate understanding of the important subtleties involved in chemokine–HS interactions. An exciting avenue of research is investigating the role of 3‐O sulphation in chemokine–HS interactions. The tools to analyse the role of 3‐O sulphation are beginning to emerge, allowing the development of our knowledge of this overlooked HS modification.^73^

N‐, 2‐O, 3‐O and 6‐O sulphation tune the ability of HS GAGs to bind to specific chemokines. Removal of the enzymes that drive HS GAG sulphation, sulphotransferases, affects leukocyte recruitment.^83^, ^84^, ^85^ These findings demonstrate the importance of HS GAG sulphation in the immune response; it is, therefore, important to consider their regulation in different biological scenarios including inflammation and disease. The nature of GAG sulphation has been shown to be tissue‐specific.^86^ Expression of sulphotransferases that produce differential GAG sulphation is complex but has been shown to be stimulus specific during cytokine‐driven inflammation.^87^, ^88^ Future studies are needed to de‐tune changes in GAG sulphation during inflammation and investigate how this regulates chemokine‐mediated leukocyte recruitment. For example, recent literature has demonstrated and explored the biological role of enhanced 2‐O sulphation in HS fragments found in the serum of individuals with sepsis.^89^

Although we are still at the beginning of understanding the role of chemokine–GAG interactions in biological events, it is becoming apparent that this may be a key player in the specificity of the chemokine system. Given the fine‐tuning that is possible within GAG structure, seen in a range of different scenarios, we can easily imagine that certain tissues or locations may produce GAGs that favour interactions with specific chemokines, as required, for local effects. Indeed, this may be important for either retention or long‐distance diffusion of chemokines that are produced at sites of inflammation but that act far away, e.g. CCL7 that mediates monocyte egress from the bone marrow.^33^, ^74^

The challenge of advancing this aspect of chemokine biology will be to combine rapidly developing knowledge of GAG biochemistry with analysis of how GAG fine structure facilitates chemokine function in biological and disease settings. In particular, recent moves towards being able to detune these interactions and sequence isolated GAG structures will revolutionize our understanding of GAGs in biological scenarios.^90^, ^91^, ^92^

## Conclusion

The idea of redundancy of the chemokine system was largely driven by early *in vitro* studies demonstrating that multiple chemokines can drive chemotaxis of the same cell types through overlapping receptors. However, as previously hypothesized elsewhere, in‐depth studies designed to dissect this redundancy phenomenon in biological scenarios have demonstrated that the system is built on specificity of function. In‐depth analysis has begun to reveal that each chemokine receptor and ligand plays a specific role in facilitating the immune response.

These studies and a large body of supporting *in vitro* findings, have revealed an array of mechanisms that facilitate this specificity: primarily differential expression of ligands and receptors in combination with biased signalling. The specific nature of chemokine–GAG interactions is emerging as a further key mechanism that facilitates the specificity of the chemokine system.

Now that specificity of function is being established, the challenge is to undertake subtle analysis to determine which chemokines and receptors are integral at specific times and locations during inflammatory disease, particularly using human samples. Only then will we really be able to ameliorate chemokine‐driven disease by informed and intelligent target selection.

## Disclosures

The author has no conflict of interest.
